# Data vs Dogma in Peyronie's Disease

**DOI:** 10.1590/S1677-5538.IBJU.2016.06.02

**Published:** 2016

**Authors:** Ryan P. Terlecki, Alison M. Rasper

**Affiliations:** 1 Wake Forest Baptist Health, NY, USA

Curvature of the erect penis from elements of internal fibrosis has been recognized for centuries (first described in 1743), yet our understanding still seems limited. Guidelines exist in both the United States and Europe, with most based on low level evidence and opinion (1, 2). Men afflicted by this situation are typically lumped together and labeled with the singular descriptor of Peyronie's disease. The level of evidence for the pathophysiology and natural history of this affliction is poor, as is the awareness of data surrounding treatment modalities. This is evidenced by the fact that one of the most commonly provided interventions is Vitamin E, which has not been shown to provide benefit and is not recommended by existing guidelines. In medical practice, it is dangerous to equate shared assumptions with fact, as this may limit pursuit of additional knowledge. Additionally, in the absence of evidence, logic should prevail.

The moniker of Peyronie's disease implies that the patient is clinically affected (thus a state of disease) rather than palpable, yet asymptomatic induration of the penile shaft. This term is used by providers to describe anatomical distortion of the penis from fibrosis. The concept of genetic predisposition has been suggested based on concomitant appreciation with Dupuytren's contracture, but this is unproven. As retrospective studies consistently note that the most common etiology is idiopathic, and considering that patient presentations are variable, it seems likely that there are multiple sets of circumstances that may produce this symptom complex rather than all men manifesting a shared underlying condition.

The fibrosis seen in many patients is similar to the injury response seen in other parts of the body. Consider the familiar sounding tale of myositis ossificans whereby repetitive trauma leads to ectopic calcification within a muscle. King provides a detailed description that involves an acute and mature phase, “loss of stretch”, and the “potential to cause pain and loss of function” (3). Stretch therapy has not been found to be helpful for this condition, and agents like indomethacin have been used in the absence of evidence. The concept has been put forth that recurrent injury results from premature return to activity. The similarities between such description and clinical experience with Peyronie's disease should be obvious to the sexual health professional.

A commonly suggested mechanism for development of a penile plaque is repetitive microtrauma from sexual activity that results in disruption of the tunica albuginea followed by a dysregulated healing response culminating in excess collagen deposition (1, 2). This seems problematic based on logic. If one considers the usual scenario of heterosexual vaginal intercourse, the most commonly applied forces to the penis result in dorsal flexion. In this state, the convex (i.e., ventral) side has increased tension, whereas the concave (i.e., dorsal) side has decreased tension. This explains why corporal disruptions in penile fractures are commonly noted on the ventral tunica, and this phenomenon is analogous to a greenstick fracture in children. Thus, it would seem more logical that the process may be due to damaged cavernosal tissue that induces a fibrotic reaction that secondarily involves the overlying tunica albuginea, akin to activating a ‘glow-stick’.

Occasionally, men will be seen with ventral curvature that is not congenital in nature. Some will describe sudden development after being discharged after a hospitalization involving an indwelling Foley catheter. It is conceivable that patients may have unconscious erections despite catheterization, and that the penis may not extend in a natural and comfortable manner with a firmly secured catheter dry enough to where friction precludes sliding of the urethra over the surface. Likewise, I have seen patients present with ventral curvature following aggressive direct vision internal urethrotomy of bulbar strictures. Despite the spongiosum being thinnest at 12 o'clock, many providers still insist on cutting at this location during DVIU, often to the point of observed bleeding. This may easily represent passage through the tunica of the urethra to the level of the septum. So, while these men may have iatrogenically induced fibrosis within the penis, once curvature develops, they will be classified as having Peyronie's disease.

The dogmatic approach to timing of intervention with Peyronie's disease has been to wait for resolution of pain and stabilization of curvature. It seems logical to assume that the pain is secondary to inflammation, common to the wound-healing response. It also seems somewhat logical that one wouldn't want to surgically intervene for curvature if there was a reasonable chance that further curvature was inevitable and could be better managed by delaying the operation. However, once a penis begins to take on curvature, most commonly dorsal in nature, the penis would seem more prone to flexion based trauma. Similarly, patients with less turgid erections are more subject to such forces and resultant elastic tissue fatigue that may lead to additional rupture of fibers and multiple scars. Thus, it could also reasonably be asserted that earlier stabilization of curvature may limit the extent of damage and the ultimate degree of distortion. Whether this would be best accomplished by plication or use of PDE5Is to improve rigidity and limit the flexibility of the erect penis is unclear. Also, research evaluating the therapeutic potential of PDE5Is based on antifibrotic properties seems confounded by the benefit of improved axial stability. The AUA guidelines state that “surgical outcomes for patients with active disease are not known” (statement 17) (1). If one considers the situation of penile fracture, it is generally regarded that non-operative management is ill-advised. Although perhaps not as clinically severe or acute in presentation, the ongoing injury involved in stimulating penile plaque formation may exist along the same spectrum.

Diagnosis of a problem can be made based on a patient's history. The degree of the problem can be characterized by physical examination and documentation of the level of curvature. Although penile duplex studies are used by some providers, there is no evidence that the results change physician decision-making, although it is conceivable that it may affect patient perception.

Within the AUA guidelines, it is important to note that no treatments options are supported by Grade A evidence. Of the available treatment modalities, the only agent approved by the FDA is collagenase Clostridium histolyticum (CCH). This is felt to be an option for patients with stable dorsal curvature of 30-90 degrees in the absence of erectile dysfunction. The guidelines state that intralesional interferon or verapamil can be considered, but notes that the “evidence for efficacy is weak”. In vitro data shows that verapamil decreases fibroblast proliferation and encourages degradation of collagen, rather than production. Thus, proponents of intralesional injection (ILI) argue that it is “reasonable and scientifically sensible” and some data has shown reduced pain and curvature in 30-60% of men after 12 injections over 6 months (4). However, the data is quite poor. Russell et al reviewed the literature in 2006, noting 19 studies, 17 of which reported positive results (5). These studies included use of steroids, CCH, verapamil, and interferon. Oxford criteria was applied to grade the evidence from strongest (1) to weakest (5), and 16/19 (84%) were level 4, with the only level 1 study involving a multicenter study of interferon that found no improvement in sexual function (6).

It seems that if the positive results are to be believed, the common denominator in these studies is not the drug, but the needle. Consider the outcomes with normal saline (NS), which has shown statistically significant improvement in curvature, with nicardipine doing no better (7). When compared to verapamil in electromotive therapy in a double blinded study, there was no significant difference (8). In the only level 1 study on ILI mentioned earlier, NS showed statistically significant improvements in curvature, plaque size, and plaque density (6). In nearly all of the articles published, full disclosure of injection technique is lacking. In fact, many providers will pass the needle back and forth through the plaque numerous times, essentially rendering this minimally-invasive surgery by needle fracture. Given the absence of ultrasound guidance in most cases and the practical limitations of using a small gauge needle to force fluid to spread through an extremely dense (and occasionally calcified) laminar plaque, it is not surprising that we have demonstrated to senior providers (with ultrasound) that when they thought the needle was inside the plaque, it was well within the corpora cavernosum.

CCH was approved for ILI based on two randomized, multicenter, double-blind placebo-controlled trials involving 832 patients with dorsal curvature, no calcifications, and ‘stable’ disease (9). On average, patients were 57 y/o, with 4 years of curvature that averaged 50 degrees, and 50% had ED. Comparing baseline to patient status at 52 weeks, patients treated with CCH improved from 48.8 to 31 degrees, and those given NS improved from 49 to 39 degrees. In the second study, the CCH group improved from 51.3 to 35.1 degrees, while the NS group improved from 49.6 to 41.1 degrees. The difference was reported as statistically significant, but this was calculated based on percent change and not the absolute improvement in number of degrees. Perspective seems important here. Many experts have identified 30 degrees as the point of problematic curvature, which is reflected in the trial design (but may also be somewhat dogmatic). At the end of both trials, both arms still had a final average curvature greater than 30 degrees. Additionally, CCH reduced curvature by 17% more than NS in Study 1 and 11% in Study 2. With the average curvature of about 50 degrees, this amounts to an additional improvement of 5.5-8.5 degrees for $26,429 in the US, which is 9x more expensive than plication (10). Also, if one looks closely at the 2nd study, the improvement bottoms out at 42 weeks and starts worsening again by 52 weeks (end of study). This suggests the possibility that patient curvature may be returning toward baseline. Data was presented at the 2016 AUA Annual Meeting on 78 patients who completed 4 cycles of CCH, with curvature changing from 58.5 to 42.0 degrees (11). Only 6/78 men had restoration of the ability to engage in intercourse and 33% had glans hypoesthesia. In the initial studies, 92% of patients had at least one adverse reaction, but corporal rupture was rare (9). However, a recent survey of urologists noted that 34% had witnessed this complication in practice, typically around 5 days after the last CCH injection (12).

The threats to male sexual function are numerous. Nearly half of men with PD have mild or moderate depression (13). Erectile function typically worsens with age, and time stops for no man. The panel for the AUA guidelines asserted that ineffective therapies that postpone return to sexual activity places a moderate burden on the patient (1). For a man eager to return to regular sexual activity, a therapeutic approach that involves a year of his life and is unlikely to result in a state that experts define as below a problematic cutoff seems ill-advised. Dogma should be perpetually challenged in medicine. In regard to PD, despite traditional mantra advocating stability of curvature before correction, a logical argument can be made for early intervention and restoration of rigidity. The often asserted mechanism of dorsal tunical disruption as the initiator of disease should be questioned. Transparency is needed in descriptions of technique related to ILI, and NS should no longer be considered a placebo and ILI should be viewed as surgical therapy via needle fracture. CCH therapy, when viewed with practical perspective, seems to be of limited value. Cease and desist letters may be need to stop providers from using ineffective topical and oral therapies.
